# Extraordinary Feat

**Published:** 1855-01

**Authors:** 


*Extraordinary Feat.*—On Wednesday, at 4 o'clock, P. M., Mr. William Wheeler, "the celebrated Western Walker," commenced the task of walking one hundred and one consecutive hours; and, inasmuch as it was alleged that a similar feat was not accomplished, particular pains were taken by those interested that there might be no humbug in the present instance. The first night was passed very comfortably; and during the next night and day he evinced no signs of fatigue. On Friday he appeared to feel the effects of a lack of rest, and his limbs commenced swelling. He was watched closely that night, and at times his mind wandered, and he was unconscious of anything going on near him. The next day (Saturday) he recovered his wonted vigor, and, with the encouragement of his friends near by, seemed quite fresh. His limbs, too, which bore evidence of his endurance, improved in appearance, and the approach of last night was met with much confidence by himself and others. He continued to walk, but with difficulty. His constitution required all the stimulus that consistent with his safety could be given. He became delirious, and had to be guarded to prevent him leaving the plank, and, if possible, the room. On Sunday morning the swelling commenced again, and the day passed tediously. Large crowds visited him, which did much to cheer him on. In the evening the audience became literally packed, and at one time it was seriously proposed clearing the room for the purpose of giving the pedestrian air. At 9 o'clock and 9 minutes, it was announced by Dr. Rowell, his physician, that his time was up. He left the plank, and walked through the crowd with perfect composure. Three cheers were given for him, which were duly acknowledged. This proved that he was perfectly sensible, and which, under these circumstances, was looked upon as a strange occurrence. A carriage being in waiting, he entered it, and immediately fell into a sound slumber. On arriving at the destination of the vehicle, he awoke, and, with a firm tread, went into the house, and, after partaking of such nourishment as was provided for him, retired to bed. The only food or drink allowed him during the time of this extraordinary performance was beef tea, (very strong,) raw eggs, brandy, and wine. This is the greatest feat of the kind on record.—*New York Tribune, from San Francisco Paper.*

